# Supplementary material for: Antiproliferative and Cytotoxic Properties of Propynoyl Betulin Derivatives against Human Ovarian Cancer Cells: In Vitro Studies
Source: Int J Mol Sci. 2023 Nov 18;24(22):16487. doi: 10.3390/ijms242216487 (PMC10671498; doi:10.3390/ijms242216487)
Supplement: Supplementary file 1 [file ijms-24-16487-s001.zip › ijms-2682581-supplementary.pdf]

Supplementary — Statistical Anaylysis

Supplementary Table S1. Statistical analysis of the data presented in Figure 2 which refers to SK-OV-3 cells proliferation curve after exposition to compound 1, performed using the Kruskal-Wallis test. The p-value is given in the table. Values marked in red indicate statistically significant differences (significance level p<0.05).

**24 hours**							
	**Control**	**0.1 μM**	**0.3 μM**	**1 μM**	**3 μM**	**10 μM**	**30 μM**
Control		1.0000	1.0000	1.0000	1.0000	0.0002	0.0012
0.1 μM	1.0000		1.0000	1.0000	1.0000	0.0030	0.0126
0.3 μM	1.0000	1.0000		1.0000	1.0000	0.0014	0.0063
1 μM	1.0000	1.0000	1.0000		1.0000	0.0357	0.1178
3 μM	1.0000	1.0000	1.0000	1.0000		0.0028	0.0115
10 μM	0.0002	0.0030	0.0014	0.0357	0.0028		1.0000
30 μM	0.0012	0.0126	0.0063	0.1178	0.0115	1.0000	
**72 hours**							
	**Control**	**0.1 μM**	**0.3 μM**	**1 μM**	**3 μM**	**10 μM**	**30 μM**
Control		1.0000	1.0000	1.0000	1.0000	0.0012	0.0007
0.1 μM	1.0000		1.0000	1.0000	1.0000	0.0005	0.0003
0.3 μM	1.0000	1.0000		1.0000	1.0000	0.0005	0.0003
1 μM	1.0000	1.0000	1.0000		1.0000	0.0003	0.0001
3 μM	1.0000	1.0000	1.0000	1.0000		0.0019	0.0011
10 μM	0.0012	0.0005	0.0005	0.0003	0.0019		1.0000
30 μM	0.0007	0.0003	0.0003	0.0001	0.0011	1.0000	
**120 hours**							
	**Control**	**0.1 μM**	**0.3 μM**	**1 μM**	**3 μM**	**10 μM**	**30 μM**
Control		1.0000	1.0000	1.0000	0.4294	0.0001	0.0000
0.1 μM	1.0000		1.0000	1.0000	0.2302	0.0000	0.0000
0.3 μM	1.0000	1.0000		1.0000	0.5952	0.0002	0.0000
1 μM	1.0000	1.0000	1.0000		1.0000	0.0005	0.0001
3 μM	0.4294	0.2302	0.5952	1.0000		0.4691	0.2247
10 μM	0.0001	0.0000	0.0002	0.0005	0.4691		1.0000
30 μM	0.0000	0.0000	0.0000	0.0001	0.2247	1.0000	

Supplementary Table S2. Statistical analysis of the data presented in Figure 2 which refers to SK-OV-3 cells proliferation curve after exposition to compound **2**, performed using the Kruskal-Wallis test. The *p*-value is given in the table. Values marked in red indicate statistically significant differences (significance level $p < 0.05$).

**24 hours**							
	**Control**	**0.1 μM**	**0.3 μM**	**1 μM**	**3 μM**	**10 μM**	**30 μM**

Control		1.0000	0.0338	0.0000	0.0000	0.0000	0.0000
0.1 µM	1.0000		1.0000	0.0028	0.0003	0.0001	0.0023
0.3 µM	0.0338	1.0000		0.5010	0.1104	0.0544	0.4342
1 µM	0.0000	0.0028	0.5010		1.0000	1.0000	1.0000
3 µM	0.0000	0.0003	0.1104	1.0000		1.0000	1.0000
10 µM	0.0000	0.0001	0.0544	1.0000	1.0000		1.0000
30 µM	0.0000	0.0023	0.4342	1.0000	1.0000	1.0000	
**72 hours**							
	**Control**	**0.1 µM**	**0.3 µM**	**1 µM**	**3 µM**	**10 µM**	**30 µM**
Control		1.0000	0.0049	0.0002	0.0000	0.0000	0.0000
0.1 µM	1.0000		0.0388	0.0301	0.0001	0.0003	0.0001
0.3 µM	0.0049	0.0388		0.9941	0.0108	0.0373	0.0110
1 µM	0.0002	0.0301	0.9941		1.0000	1.0000	1.0000
3 µM	0.0000	0.0001	0.0108	1.0000		1.0000	1.0000
10 µM	0.0000	0.0003	0.0373	1.0000	1.0000		1.0000
30 µM	0.0000	0.0001	0.0110	1.0000	1.0000	1.0000	
**120 hours**							
	**Control**	**0.1 µM**	**0.3 µM**	**1 µM**	**3 µM**	**10 µM**	**30 µM**
Control		1.0000	0.0087	0.0043	0.0000	0.0000	0.0000
0.1 µM	1.0000		0.0318	0.0219	0.0000	0.0000	0.0001
0.3 µM	0.0087	0.0318		1.0000	0.0068	0.0049	0.0121
1 µM	0.0043	0.0219	1.0000		0.4955	0.3927	0.7266
3 µM	0.0000	0.0000	0.0068	0.4955		1.0000	1.0000
10 µM	0.0000	0.0000	0.0049	0.3927	1.0000		1.0000
30 µM	0.0000	0.0001	0.0121	0.7266	1.0000	1.0000	

Supplementary Table S3. Statistical analysis of the data presented in Figure 2 which refers to SK-OV-3 cells proliferation curve after exposition to compound **3**, performed using the Kruskal-Wallis test. The *p*-value is given in the table. Values marked in red indicate statistically significant differences (significance level $p < 0.05$).

**24 hours**							
	**Control**	**0.1 µM**	**0.3 µM**	**1 µM**	**3 µM**	**10 µM**	**30 µM**
Control		1.0000	0.7342	0.0000	0.0000	0.0000	0.0001
0.1 µM	1.0000		1.0000	0.0034	0.0000	0.0001	0.0049
0.3 µM	0.7342	1.0000		0.1850	0.0018	0.0117	0.2443
1 µM	0.0000	0.0034	0.1850		1.0000	1.0000	1.0000

3 μM	0.0000	0.0000	0.0018	1.0000		1.0000	1.0000
10 μM	0.0000	0.0001	0.0117	1.0000	1.0000		1.0000
30 μM	0.0001	0.0049	0.2443	1.0000	1.0000	1.0000	
**72 hours**							
	**Control**	**0.1 μM**	**0.3 μM**	**1 μM**	**3 μM**	**10 μM**	**30 μM**
Control		1.0000	0.9181	0.0357	0.0000	0.0000	0.0000
0.1 μM	1.0000		1.0000	0.1062	0.0000	0.0000	0.0000
0.3 μM	0.9181	1.0000		1.0000	0.0094	0.0095	0.0045
1 μM	0.0357	0.1062	1.0000		0.3546	0.3587	0.2065
3 μM	0.0000	0.0000	0.0094	0.3546		1.0000	1.0000
10 μM	0.0000	0.0000	0.0095	0.3587	1.0000		1.0000
30 μM	0.0000	0.0000	0.0045	0.2065	1.0000	1.0000	
**120 hours**							
	**Control**	**0.1 μM**	**0.3 μM**	**1 μM**	**3 μM**	**10 μM**	**30 μM**
Control		1.0000	1.0000	0.0474	0.0000	0.0000	0.0000
0.1 μM	1.0000		1.0000	0.0624	0.0000	0.0000	0.0000
0.3 μM	1.0000	1.0000		1.0000	0.0064	0.0048	0.0081
1 μM	0.0474	0.0624	1.0000		0.3388	0.2750	0.4016
3 μM	0.0000	0.0000	0.0064	0.3388		1.0000	1.0000
10 μM	0.0000	0.0000	0.0048	0.2750	1.0000		1.0000
30 μM	0.0000	0.0000	0.0081	0.4016	1.0000	1.0000	

Supplementary Table S4. Statistical analysis of the data presented in Figure 2 which refers to SK-OV-3 cells proliferation curve after exposition to compound **4**, performed using the Kruskal-Wallis test. The *p*-value is given in the table. Values marked in red indicate statistically significant differences (significance level $p < 0.05$).

24 hours							
	**Control**	**0.1 µM**	**0.3 µM**	**1 µM**	**3 µM**	**10 µM**	**30 µM**
Control		1.0000	1.0000	0.6478	0.0005	0.0000	0.0000
0.1 µM	1.0000		1.0000	1.0000	0.0029	0.0000	0.0000
0.3 µM	1.0000	1.0000		1.0000	0.0015	0.0000	0.0000
1 µM	0.6478	1.0000	1.0000		0.8558	0.0245	0.0607
3 µM	0.0005	0.0029	0.0015	0.8558		1.0000	1.0000
10 µM	0.0000	0.0000	0.0000	0.0245	1.0000		1.0000
30 µM	0.0000	0.0000	0.0000	0.0607	1.0000	1.0000	
**72 hours**							
	**Control**	**0.1 µM**	**0.3 µM**	**1 µM**	**3 µM**	**10 µM**	**30 µM**
Control		1.0000	1.0000	0.7495	0.0130	0.0001	0.0000
0.1 µM	1.0000		1.0000	0.3927	0.0050	0.0000	0.0000
0.3 µM	1.0000	1.0000		0.1148	0.0009	0.0000	0.0000
1 µM	0.7495	0.3927	0.1148		1.0000	0.1943	0.0199
3 µM	0.0130	0.0050	0.0009	1.0000		1.0000	0.9941
10 µM	0.0001	0.0000	0.0000	0.1943	1.0000		1.0000
30 µM	0.0000	0.0000	0.0000	0.0199	0.9941	1.0000	
**120 hours**							
	**Control**	**0.1 µM**	**0.3 µM**	**1 µM**	**3 µM**	**10 µM**	**30 µM**
Control		1.0000	1.0000	0.3311	0.0062	0.0000	0.0000
0.1 µM	1.0000		1.0000	0.3273	0.0061	0.0000	0.0000
0.3 µM	1.0000	1.0000		0.3466	0.0066	0.0000	0.0000
1 µM	0.3311	0.3273	0.3466		1.0000	0.0389	0.0754
3 µM	0.0062	0.0061	0.0066	1.0000		1.0000	1.0000
10 µM	0.0000	0.0000	0.0000	0.0389	1.0000		1.0000
30 µM	0.0000	0.0000	0.0000	0.0754	1.0000	1.0000	

Supplementary Table S5. Statistical analysis of the data presented in Figure 2 which refers to SK-OV-3 cells proliferation curve after exposition to compound **5**, performed using the Kruskal-Wallis test. The *p*-value is given in the table. Values marked in red indicate statistically significant differences (significance level $p < 0.05$).

**24 hours**							
	**Control**	**0.1 µM**	**0.3 µM**	**1 µM**	**3 µM**	**10 µM**	**30 µM**
Control		1.0000	1.0000	0.0002	0.0000	0.0004	0.0000
0.1 µM	1.0000		1.0000	0.0007	0.0000	0.0014	0.0000
0.3 µM	1.0000	1.0000		0.2623	0.0016	0.4107	0.0238
1 µM	0.0002	0.0007	0.2623		1.0000	1.0000	1.0000
3 µM	0.0000	0.0000	0.0016	1.0000		1.0000	1.0000
10 µM	0.0004	0.0014	0.4107	1.0000	1.0000		1.0000
30 µM	0.0000	0.0000	0.0238	1.0000	1.0000	1.0000	
**72 hours**							
	**Control**	**0.1 µM**	**0.3 µM**	**1 µM**	**3 µM**	**10 µM**	**30 µM**
Control		1.0000	0.8472	0.0001	0.0000	0.0000	0.0000
0.1 µM	1.0000		1.0000	0.0005	0.0002	0.0000	0.0000
0.3 µM	0.8472	1.0000		0.1636	0.0873	0.0337	0.0159
1 µM	0.0001	0.0005	0.1636		1.0000	1.0000	1.0000
3 µM	0.0000	0.0002	0.0873	1.0000		1.0000	1.0000
10 µM	0.0000	0.0000	0.0337	1.0000	1.0000		1.0000
30 µM	0.0000	0.0000	0.0159	1.0000	1.0000	1.0000	
**120 hours**							
	**Control**	**0.1 µM**	**0.3 µM**	**1 µM**	**3 µM**	**10 µM**	**30 µM**
Control		1.0000	1.0000	0.0000	0.0003	0.0000	0.0001
0.1 µM	1.0000		1.0000	0.0000	0.0001	0.0000	0.0000
0.3 µM	1.0000	1.0000		0.0208	0.1783	0.0221	0.0920
1 µM	0.0000	0.0000	0.0208		1.0000	1.0000	1.0000
3 µM	0.0003	0.0001	0.1783	1.0000		1.0000	1.0000
10 µM	0.0000	0.0000	0.0221	1.0000	1.0000		1.0000
30 µM	0.0001	0.0000	0.0920	1.0000	1.0000	1.0000	

Supplementary Table S6. Statistical analysis of the data presented in Figure 2 which refers to SK-OV-3 cells proliferation curve after exposition to compound **6**, performed using the Kruskal-Wallis test. The *p*-value is given in the table. Values marked in red indicate statistically significant differences (significance level $p < 0.05$).

**24 hours**							
	**Control**	**0.1 µM**	**0.3 µM**	**1 µM**	**3 µM**	**10 µM**	**30 µM**
Control		1.0000	0.5523	0.0000	0.0001	0.0001	0.0000
0.1 µM	1.0000		1.0000	0.0000	0.0022	0.0037	0.0016
0.3 µM	0.5523	1.0000		0.0018	0.2718	0.3839	0.2141
1 µM	0.0000	0.0000	0.0018		1.0000	1.0000	1.0000
3 µM	0.0001	0.0022	0.2718	1.0000		1.0000	1.0000
10 µM	0.0001	0.0037	0.3839	1.0000	1.0000		1.0000
30 µM	0.0000	0.0016	0.2141	1.0000	1.0000	1.0000	
**72 hours**							
	**Control**	**0.1 µM**	**0.3 µM**	**1 µM**	**3 µM**	**10 µM**	**30 µM**
Control		1.0000	1.0000	0.0000	0.0005	0.0001	0.0000
0.1 µM	1.0000		1.0000	0.0000	0.0005	0.0001	0.0000
0.3 µM	1.0000	1.0000		0.0016	0.3349	0.1239	0.0724
1 µM	0.0000	0.0000	0.0016		1.0000	1.0000	1.0000
3 µM	0.0005	0.0005	0.3349	1.0000		1.0000	1.0000
10 µM	0.0001	0.0001	0.1239	1.0000	1.0000		1.0000
30 µM	0.0000	0.0000	0.0724	1.0000	1.0000	1.0000	
**120 hours**							
	**Control**	**0.1 µM**	**0.3 µM**	**1 µM**	**3 µM**	**10 µM**	**30 µM**
Control		1.0000	0.3971	0.0000	0.0000	0.0000	0.0004
0.1 µM	1.0000		1.0000	0.0000	0.0005	0.0004	0.0296
0.3 µM	0.3971	1.0000		0.0007	0.0686	0.0515	1.0000
1 µM	0.0000	0.0000	0.0007		1.0000	1.0000	0.5405
3 µM	0.0000	0.0005	0.0686	1.0000		1.0000	1.0000
10 µM	0.0000	0.0004	0.0515	1.0000	1.0000		1.0000
30 µM	0.0004	0.0296	1.0000	0.5405	1.0000	1.0000	

Supplementary Table S7. Statistical analysis of the data presented in Figure 3 which refers to OVCAR-3 cells proliferation curve after exposition to compound **1**, performed using the Kruskal-Wallis test. The *p*-value is given in the table. Values marked in red indicate statistically significant differences (significance level $p < 0.05$).

**24 hours**							
	**Control**	**0.1 μM**	**0.3 μM**	**1 μM**	**3 μM**	**10 μM**	**30 μM**
Control		1.0000	1.0000	1.0000	1.0000	1.0000	0.4796
0.1 μM	1.0000		1.0000	1.0000	1.0000	1.0000	0.2274
0.3 μM	1.0000	1.0000		1.0000	1.0000	1.0000	0.2593
1 μM	1.0000	1.0000	1.0000		1.0000	1.0000	0.0143
3 μM	1.0000	1.0000	1.0000	1.0000		1.0000	0.2220
10 μM	1.0000	1.0000	1.0000	1.0000	1.0000		0.0055
30 μM	0.4796	0.2274	0.2593	0.0143	0.2220	0.0055	
**72 hours**							
	**Control**	**0.1 μM**	**0.3 μM**	**1 μM**	**3 μM**	**10 μM**	**30 μM**
Control		1.0000	0.9000	1.0000	0.4691	0.0896	0.0000
0.1 μM	1.0000		1.0000	1.0000	1.0000	0.5643	0.0001
0.3 μM	0.9000	1.0000		1.0000	1.0000	1.0000	0.0021
1 μM	1.0000	1.0000	1.0000		1.0000	1.0000	0.0006
3 μM	0.4691	1.0000	1.0000	1.0000		1.0000	0.0060
10 μM	0.0896	0.5643	1.0000	1.0000	1.0000		0.3628
30 μM	0.0000	0.0001	0.0021	0.0006	0.0060	0.3628	
**120 hours**							
	**Control**	**0.1 μM**	**0.3 μM**	**1 μM**	**3 μM**	**10 μM**	**30 μM**
Control		1.0000	1.0000	1.0000	1.0000	0.0087	0.0010
0.1 μM	1.0000		1.0000	1.0000	1.0000	0.0036	0.0000
0.3 μM	1.0000	1.0000		1.0000	1.0000	0.0284	0.0002
1 μM	1.0000	1.0000	1.0000		1.0000	0.0011	0.0000
3 μM	1.0000	1.0000	1.0000	1.0000		0.0095	0.0001
10 μM	0.0087	0.0036	0.0284	0.0011	0.0095		1.0000
30 μM	0.0010	0.0000	0.0002	0.0000	0.0001	1.0000	

Supplementary Table S8. Statistical analysis of the data presented in Figure 3 which refers to OVCAR-3 cells proliferation curve after exposition to compound **2**, performed using the Kruskal-Wallis test. The *p*-value is given in the table. Values marked in red indicate statistically significant differences (significance level $p < 0.05$).

**24 hours**							
	**Control**	**0.1 µM**	**0.3 µM**	**1 µM**	**3 µM**	**10 µM**	**30 µM**
Control		1.0000	0.0167	0.0020	0.0019	0.0000	0.0000
0.1 µM	1.0000		0.2090	0.0027	0.0025	0.0000	0.0000
0.3 µM	0.0167	0.2090		1.0000	1.0000	0.3796	0.0724
1 µM	0.0020	0.0027	1.0000		1.0000	1.0000	1.0000
3 µM	0.0019	0.0025	1.0000	1.0000		1.0000	1.0000
10 µM	0.0000	0.0000	0.3796	1.0000	1.0000		1.0000
30 µM	0.0000	0.0000	0.0724	1.0000	1.0000	1.0000	
**72 hours**							
	**Control**	**0.1 µM**	**0.3 µM**	**1 µM**	**3 µM**	**10 µM**	**30 µM**
Control		1.0000	0.0247	0.0000	0.0000	0.0000	0.0000
0.1 µM	1.0000		0.0020	0.0001	0.0002	0.0004	0.0049
0.3 µM	0.0247	0.0020		0.0362	0.0607	0.0839	0.4955
1 µM	0.0000	0.0001	0.0362		1.0000	1.0000	1.0000
3 µM	0.0000	0.0002	0.0607	1.0000		1.0000	1.0000
10 µM	0.0000	0.0004	0.0839	1.0000	1.0000		1.0000
30 µM	0.0000	0.0049	0.4955	1.0000	1.0000	1.0000	
**120 hours**							
	**Control**	**0.1 µM**	**0.3 µM**	**1 µM**	**3 µM**	**10 µM**	**30 µM**
Control		1.0000	0.0000	0.0000	0.0000	0.0000	0.0000
0.1 µM	1.0000		0.0000	0.0000	0.0001	0.0002	0.0009
0.3 µM	0.0000	0.0000		0.0218	0.0442	0.0501	0.1656
1 µM	0.0000	0.0000	0.0218		1.0000	1.0000	1.0000
3 µM	0.0000	0.0001	0.0442	1.0000		1.0000	1.0000
10 µM	0.0000	0.0002	0.0501	1.0000	1.0000		1.0000
30 µM	0.0000	0.0009	0.1656	1.0000	1.0000	1.0000	

Supplementary Table S9. Statistical analysis of the data presented in Figure 3 which refers to OVCAR-3 cells proliferation curve after exposition to compound **3**, performed using the Kruskal-Wallis test. The *p*-value is given in the table. Values marked in red indicate statistically significant differences (significance level $p < 0.05$).

**24 hours**							
	**Control**	**0.1 μM**	**0.3 μM**	**1 μM**	**3 μM**	**10 μM**	**30 μM**
Control		1.0000	1.0000	0.0037	0.0001	0.0001	0.0000
0.1 μM	1.0000		1.0000	0.0071	0.0002	0.0001	0.0000
0.3 μM	1.0000	1.0000		0.2882	0.0231	0.0126	0.0003
1 μM	0.0037	0.0071	0.2882		1.0000	1.0000	1.0000
3 μM	0.0001	0.0002	0.0231	1.0000		1.0000	1.0000
10 μM	0.0001	0.0001	0.0126	1.0000	1.0000		1.0000
30 μM	0.0000	0.0000	0.0003	1.0000	1.0000	1.0000	
**72 hours**							
	**Control**	**0.1 μM**	**0.3 μM**	**1 μM**	**3 μM**	**10 μM**	**30 μM**
Control		1.0000	1.0000	0.0444	0.0011	0.0002	0.0000
0.1 μM	1.0000		1.0000	0.0114	0.0001	0.0000	0.0000
0.3 μM	1.0000	1.0000		0.4391	0.0010	0.0002	0.0000
1 μM	0.0444	0.0114	0.4391		1.0000	0.7651	0.0318
3 μM	0.0011	0.0001	0.0010	1.0000		1.0000	1.0000
10 μM	0.0002	0.0000	0.0002	0.7651	1.0000		1.0000
30 μM	0.0000	0.0000	0.0000	0.0318	1.0000	1.0000	
**120 hours**							
	**Control**	**0.1 μM**	**0.3 μM**	**1 μM**	**3 μM**	**10 μM**	**30 μM**
Control		1.0000	1.0000	0.0041	0.0001	0.0001	0.0000
0.1 μM	1.0000		1.0000	0.0017	0.0000	0.0000	0.0000
0.3 μM	1.0000	1.0000		0.0677	0.0024	0.0039	0.0017
1 μM	0.0041	0.0017	0.0677		1.0000	1.0000	1.0000
3 μM	0.0001	0.0000	0.0024	1.0000		1.0000	1.0000
10 μM	0.0001	0.0000	0.0039	1.0000	1.0000		1.0000
30 μM	0.0000	0.0000	0.0017	1.0000	1.0000	1.0000	

Supplementary Table S10. Statistical analysis of the data presented in Figure 3 which refers to OVCAR-3 cells proliferation curve after exposition to compound **4**, performed using the Kruskal-Wallis test. The *p*-value is given in the table. Values marked in red indicate statistically significant differences (significance level $p < 0.05$).

**24 hours**							
	**Control**	**0.1 µM**	**0.3 µM**	**1 µM**	**3 µM**	**10 µM**	**30 µM**
Control		1.0000	1.0000	1.0000	0.0122	0.0001	0.0001
0.1 µM	1.0000		1.0000	1.0000	0.1443	0.0024	0.0021
0.3 µM	1.0000	1.0000		1.0000	0.9460	0.0332	0.0301
1 µM	1.0000	1.0000	1.0000		1.0000	0.0932	0.0850
3 µM	0.0122	0.1443	0.9460	1.0000		1.0000	1.0000
10 µM	0.0001	0.0024	0.0332	0.0932	1.0000		1.0000
30 µM	0.0001	0.0021	0.0301	0.0850	1.0000	1.0000	
**72 hours**							
	**Control**	**0.1 µM**	**0.3 µM**	**1 µM**	**3 µM**	**10 µM**	**30 µM**
Control		1.0000	1.0000	0.6478	0.0007	0.0000	0.0000
0.1 µM	1.0000		1.0000	1.0000	0.0076	0.0000	0.0000
0.3 µM	1.0000	1.0000		1.0000	0.4107	0.0019	0.0003
1 µM	0.6478	1.0000	1.0000		0.9650	0.0073	0.0012
3 µM	0.0007	0.0076	0.4107	0.9650		1.0000	0.8821
10 µM	0.0000	0.0000	0.0019	0.0073	1.0000		1.0000
30 µM	0.0000	0.0000	0.0003	0.0012	0.8821	1.0000	
**120 hours**							
	**Control**	**0.1 µM**	**0.3 µM**	**1 µM**	**3 µM**	**10 µM**	**30 µM**
Control		1.0000	1.0000	0.9555	0.0171	0.0000	0.0000
0.1 µM	1.0000		1.0000	0.3754	0.0043	0.0000	0.0000
0.3 µM	1.0000	1.0000		1.0000	0.0695	0.0001	0.0000
1 µM	0.9555	0.3754	1.0000		1.0000	0.0394	0.0288
3 µM	0.0171	0.0043	0.0695	1.0000		1.0000	1.0000
10 µM	0.0000	0.0000	0.0001	0.0394	1.0000		1.0000
30 µM	0.0000	0.0000	0.0000	0.0288	1.0000	1.0000	

Supplementary Table S11. Statistical analysis of the data presented in Figure 3 which refers to OVCAR-3 cells proliferation curve after exposition to compound **5**, performed using the Kruskal-Wallis test. The *p*-value is given in the table. Values marked in red indicate statistically significant differences (significance level $p < 0.05$).

**24 hours**							
	**Control**	**0.1 µM**	**0.3 µM**	**1 µM**	**3 µM**	**10 µM**	**30 µM**
Control		1.0000	1.0000	0.0000	0.0000	0.0002	0.0000
0.1 µM	1.0000		1.0000	0.0010	0.0005	0.0037	0.0012
0.3 µM	1.0000	1.0000		0.0015	0.0008	0.0051	0.0017
1 µM	0.0000	0.0010	0.0015		1.0000	1.0000	1.0000
3 µM	0.0000	0.0005	0.0008	1.0000		1.0000	1.0000
10 µM	0.0002	0.0037	0.0051	1.0000	1.0000		1.0000
30 µM	0.0000	0.0012	0.0017	1.0000	1.0000	1.0000	
**72 hours**							
	**Control**	**0.1 µM**	**0.3 µM**	**1 µM**	**3 µM**	**10 µM**	**30 µM**
Control		1.0000	1.0000	0.0441	0.0342	0.0199	0.0016
0.1 µM	1.0000		1.0000	0.2167	0.1239	0.0764	0.0078
0.3 µM	1.0000	1.0000		0.0995	0.0544	0.0323	0.0028
1 µM	0.0441	0.2167	0.0995		1.0000	1.0000	1.0000
3 µM	0.0342	0.1239	0.0544	1.0000		1.0000	1.0000
10 µM	0.0199	0.0764	0.0323	1.0000	1.0000		1.0000
30 µM	0.0016	0.0078	0.0028	1.0000	1.0000	1.0000	
**120 hours**							
	**Control**	**0.1 µM**	**0.3 µM**	**1 µM**	**3 µM**	**10 µM**	**30 µM**
Control		1.0000	1.0000	0.0199	0.0000	0.0000	0.0000
0.1 µM	1.0000		1.0000	0.0828	0.0000	0.0002	0.0000
0.3 µM	1.0000	1.0000		0.5523	0.0010	0.0031	0.0002
1 µM	0.0199	0.0828	0.5523		1.0000	1.0000	0.5347
3 µM	0.0000	0.0000	0.0010	1.0000		1.0000	1.0000
10 µM	0.0000	0.0002	0.0031	1.0000	1.0000		1.0000
30 µM	0.0000	0.0000	0.0002	0.5347	1.0000	1.0000	

Supplementary Table S12. Statistical analysis of the data presented in Figure 3 which refers to OVCAR-3 cells proliferation curve after exposition to compound **6**, performed using the Kruskal-Wallis test. The *p*-value is given in the table. Values marked in red indicate statistically significant differences (significance level $p < 0.05$).

**24 hours**							
	**Control**	**0.1 μM**	**0.3 μM**	**1 μM**	**3 μM**	**10 μM**	**30 μM**
Control		1.0000	1.0000	0.1078	0.0002	0.0000	0.0003
0.1 μM	1.0000		1.0000	0.1214	0.0002	0.0000	0.0003
0.3 μM	1.0000	1.0000		0.2376	0.0005	0.0001	0.0008
1 μM	0.1078	0.1214	0.2376		1.0000	0.9720	1.0000
3 μM	0.0002	0.0002	0.0005	1.0000		1.0000	1.0000
10 μM	0.0000	0.0000	0.0001	0.9720	1.0000		1.0000
30 μM	0.0003	0.0003	0.0008	1.0000	1.0000	1.0000	
**72 hours**							
	**Control**	**0.1 μM**	**0.3 μM**	**1 μM**	**3 μM**	**10 μM**	**30 μM**
Control		1.0000	1.0000	0.1133	0.0000	0.0000	0.0001
0.1 μM	1.0000		1.0000	0.5582	0.0000	0.0000	0.0007
0.3 μM	1.0000	1.0000		0.5405	0.0000	0.0000	0.0007
1 μM	0.1133	0.5582	0.5405		0.1850	0.1499	1.0000
3 μM	0.0000	0.0000	0.0000	0.1850		1.0000	1.0000
10 μM	0.0000	0.0000	0.0000	0.1499	1.0000		1.0000
30 μM	0.0001	0.0007	0.0007	1.0000	1.0000	1.0000	
**120 hours**							
	**Control**	**0.1 μM**	**0.3 μM**	**1 μM**	**3 μM**	**10 μM**	**30 μM**
Control		1.0000	1.0000	0.0714	0.0000	0.0000	0.0000
0.1 μM	1.0000		1.0000	0.0957	0.0000	0.0000	0.0000
0.3 μM	1.0000	1.0000		1.0000	0.0059	0.0006	0.0029
1 μM	0.0714	0.0957	1.0000		0.6343	0.1372	0.4016
3 μM	0.0000	0.0000	0.0059	0.6343		1.0000	1.0000
10 μM	0.0000	0.0000	0.0006	0.1372	1.0000		1.0000
30 μM	0.0000	0.0000	0.0029	0.4016	1.0000	1.0000	
